# Classification of Trauma-Associated Invasive Fungal Infections to Support Wound Treatment Decisions

**DOI:** 10.3201/eid2509.190168

**Published:** 2019-09

**Authors:** Anuradha Ganesan, Faraz Shaikh, William Bradley, Dana M. Blyth, Denise Bennett, Joseph L. Petfield, M. Leigh Carson, Justin M. Wells, David R. Tribble, Infectious Disease Clinical Research Program Trauma Infectious Disease Outcomes Study Group

**Affiliations:** The Henry M. Jackson Foundation for the Advancement of Military Medicine, Inc., Bethesda, Maryland, USA (A. Ganesan, F. Shaikh, W. Bradley, D. Bennett, M.L. Carson);; Uniformed Services University of the Health Sciences, Bethesda (A. Ganesan, F. Shaikh, W. Bradley, D. Bennett, M.L. Carson, D.R. Tribble);; Walter Reed National Military Medical Center, Bethesda (A. Ganesan, J.M. Wells);; Brooke Army Medical Center, San Antonio, Texas, USA (W. Bradley, D.M. Blyth);; Landstuhl Regional Medical Center, Landstuhl, Germany (J.L. Petfield)

**Keywords:** invasive fungal infections, wound infection, blast wound, mucormycosis, trauma, fungi, bacteria, Afghanistan

## Abstract

To evaluate a classification system to support clinical decisions for treatment of contaminated deep wounds at risk for an invasive fungal infection (IFI), we studied 246 US service members (413 wounds) injured in Afghanistan (2009–2014) who had laboratory evidence of fungal infection. A total of 143 wounds with persistent necrosis and laboratory evidence were classified as IFI; 120 wounds not meeting IFI criteria were classified as high suspicion (patients had localized infection signs/symptoms and had received antifungal medication for >10 days), and 150 were classified as low suspicion (failed to meet these criteria). IFI patients received more blood than other patients and had more severe injuries than patients in the low-suspicion group. Fungi of the order Mucorales were more frequently isolated from IFI (39%) and high-suspicion (21%) wounds than from low-suspicion (9%) wounds. Wounds that did not require immediate antifungal therapy lacked necrosis and localized signs/symptoms of infection and contained fungi from orders other than Mucorales.

## Abstract 2

The proposed classification, based on diagnostic certainty, provides a framework for determining initial empiric and subsequent targeted therapy.

Cutaneous invasive fungal infections (IFI) occur in deep tissue wounds contaminated by environmental debris; such wounds are caused by agricultural accidents, tornadoes, and blast trauma (*1**–**7*). Among severely injured trauma patients (military and civilian), IFIs have emerged as a serious complication (*2*,*4*,*6*,*7*). Specifically, coinciding with the surge of service members into Afghanistan and the rising frequency of blast injuries, IFIs have emerged as serious complications of blast trauma sustained by soldiers while on foot patrol. The first reported cases were among UK military personnel injured while in Helmand Province, Afghanistan (*8*), followed by 37 cases among US military personnel (*1*). Common characteristics among these IFI patients were battlefield blast injuries sustained while on foot patrol, extensive wounds or amputation sites heavily contaminated with debris, and receipt of large-volume blood transfusions (>8 units of blood) within 24 hours of injury (*1*,*8*). These infections were associated with substantial morbidity (e.g., surgical amputations and hemipelvectomies) and considerable death rates, especially before the syndrome was recognized (*1*,*3*,*9*). Given the progressive nature and substantial morbidity associated with such infections, patients at risk for IFI needed to be identified and given early treatment with aggressive surgical debridement and systemic antifungal therapy. Defining what constitutes a wound suspected of having an IFI is also critical, and clinicians were advised to use hallmark wound necrosis and preliminary risk factors to establish an IFI diagnosis as early as possible (*10*). 

For the initial IFI cases in the United States, the median time from injury to IFI diagnosis was 10 days. In 2011, in an effort to hasten IFI diagnoses, a performance improvement measure that involved early tissue sampling of wounds (usually after 1 debridement) from those at high risk for IFI was introduced at the Landstuhl Regional Medical Center in Germany (LRMC) (*11*). After the introduction of this diagnostic approach, it became clear that fungal contamination of battlefield blast wounds was common (*12*,*13*); therefore, it is necessary to differentiate wounds contaminated by fungi from those that are truly infected. Furthermore, the inability to easily discriminate between infected and colonized wounds (based on injury and patient demographic characteristics) led to wide practice variations. In this study, we examined the epidemiology of IFIs among US military personnel injured in Afghanistan. We also assessed the discriminatory capacity of clinical and pathologic/microbiological criteria for stratifying patients into risk groups that would enable treatment and resource prioritization and reduce practice variations.

## Methods

### Study Population

Data were collected as part of the Department of Defense, Department of Veterans Affairs, multicenter Trauma Infectious Disease Outcomes Study, an observational study of infectious complications among wounded military personnel (*14*). Eligible patients were US service members who had sustained traumatic wounds while on the battlefield in Afghanistan during June 1, 2009–December 31, 2014, and who had been evacuated to LRMC before transfer to a participating military hospital in the United States: Walter Reed National Military Medical Center (Bethesda, MD; National Naval Medical Center and Walter Reed Army Medical Center before September 2011) and Brooke Army Medical Center (San Antonio, TX). The study was approved by the institutional review board of the Uniformed Services University of the Health Sciences.

We obtained information about patient demographics, injury characteristics, trauma and clinical history, and surgical management history from the US Department of Defense Trauma Registry (*15*) and clinical laboratory results, infectious outcomes, culture and histopathology, and antifungal treatment from the Trauma Infectious Disease Outcomes Study infectious disease module (*14*). To assess patients, we used the Injury Severity Score (ISS) (*16*) and Sequential Organ Failure Assessment (SOFA) score (*17*). The ISS is an anatomic scoring system used for patients with multiple injuries. Each injury is evaluated and assigned an Abbreviated Injury Scale code, which is an anatomic consensus-based global score. The injuries are divided into 6 body regions, and the 3 most severely injured body region scores are squared and added to produce a composite score. An ISS score of 0–9 is classified as minor, 10–15 as moderate, 16–25 as severe, and >26 as critical.

### Case Definitions

We examined patients with laboratory evidence of infection with a filamentous fungus (positive histopathologic findings, positive fungal culture, or both). We modified case definitions from the 2008 European Organization for Research and Treatment of Cancer/Invasive Fungal Infections Cooperative Group and the National Institute of Allergy and Infectious Diseases Mycoses Study Group (EORTC/MSG) Consensus Group for use with trauma patients (*3*,*18*). After patients were admitted to LRMC, wounds with persistent necrosis and presence of filamentous fungus (after >2 surgical debridements) were classified as IFI (Table 1). IFI wounds were further categorized according to histopathologic findings as proven (with angioinvasion), probable (fungal hypha tissue invasion but without angioinvasion), or possible (positive cultures and negative histopathologic findings).

**Table 1:** Definitions for the classification of evidence for fungal infections*

Term	Definition†
Persistent necrosis‡	Presence of necrosis after >2 surgical debridements
Persistent laboratory evidence of fungal infection‡	Presence of positive histopathology and/or culture after >2 surgical debridements
Wounds meeting criteria for IFI	Includes wounds with persistent necrosis and persistent laboratory evidence of fungal infection
Wounds highly suspicious for fungal infection (high-suspicion wounds)	Includes wounds that did not meet the criteria for an IFI but produced signs and symptoms suggestive of a deep SSTI ascribed to a fungus (based on the use of antifungals for >10 d and a physician report). Wounds that did not meet criteria for an IFI but required a proximal amputation were included, irrespective of the duration of antifungal use.
Wounds with low suspicion for fungal Infection (low-suspicion wounds)	Includes wounds that did not meet the criteria for an IFI and did not meet the criteria for a deep SSTI. This category also includes wounds that produced signs and symptoms of a deep SSTI attributed to bacteria (based on physician report or the use of antifungals for <10 d) but with laboratory evidence of fungus (i.e., positive fungal cultures, histopathologic findings, or both).

*IFI, invasive fungal infection; SSTI, skin and soft tissue infection. †Centers for Disease Control and Prevention National Healthcare Safety Network criteria for deep SSTIs were adapted for this definition (*19*). ‡Excludes any additional debridement that was performed in the battlefield hospitals in Afghanistan.

Wounds not meeting criteria for IFI were classified as being of high or low suspicion for IFI. We modified National Healthcare Safety Network (NHSN) definitions for skin and soft tissue infections (SSTI) and used them to differentiate between high-suspicion and low-suspicion wounds. The NHSN definition of SSTI relies on the presence of localized signs and symptoms (e.g., pain, tenderness, swelling, erythema, heat) without another recognized cause (*19*). A deep SSTI met NHSN criteria and included wounds that spontaneously dehisced and those requiring surgical intervention. Wounds that met criteria for a deep SSTI that the treating physician attributed to a fungus and that were treated with antifungal medications for >10 days were classified as high-suspicion wounds. Patients who had died or undergone a definitive amputation (proximal to the infected wound) within 10 days of initiation of antifungal medication were also included because both events could lead to withdrawal of antifungal medication. The low-suspicion group included wounds that met deep SSTI criteria but were attributed by the treating physician to bacteria, wounds that failed to meet deep SSTI criteria, and deep SSTIs for which the patient received antifungal medication for <10 days.

### Statistical Considerations

Because multiple traumatic injuries were frequent, patients often had multiple wounds with laboratory evidence of a fungus. We evaluated wound characteristics (e.g., culture findings) and patient-level characteristics (e.g., injury severity). Patients with 2 wounds that met different classifications were classified according to the highest level (e.g., if 1 wound met IFI criteria and the other was of low suspicion, the patient was classified as having an IFI). We performed a restricted analysis for patients with wounds that met criteria for a single classification.

Fungal culture results were categorized into 4 main groups: all fungi belonging to the order Mucorales (with/without fungi of other genera), fungi of the genus *Aspergillus* (with/without other fungi), fungi of genus *Fusarium* (with/without other fungi), and all other fungi. Polymicrobial wounds may be counted under multiple fungal groups (e.g., order Mucorales plus *Aspergillus* spp.). Data from patients who had undergone multiple debridements and multiple specimen collections were pooled for the wound site.

We compared categorical variables by using the Fisher exact and χ^2^ tests. We compared overall continuous variable distributions by using the Kruskal-Wallis test and performed statistical analyses in SAS version 9.3 (https://www.sas.com). We defined significance as p<0.05.

## Results

### Study Population

**Figure 1:**
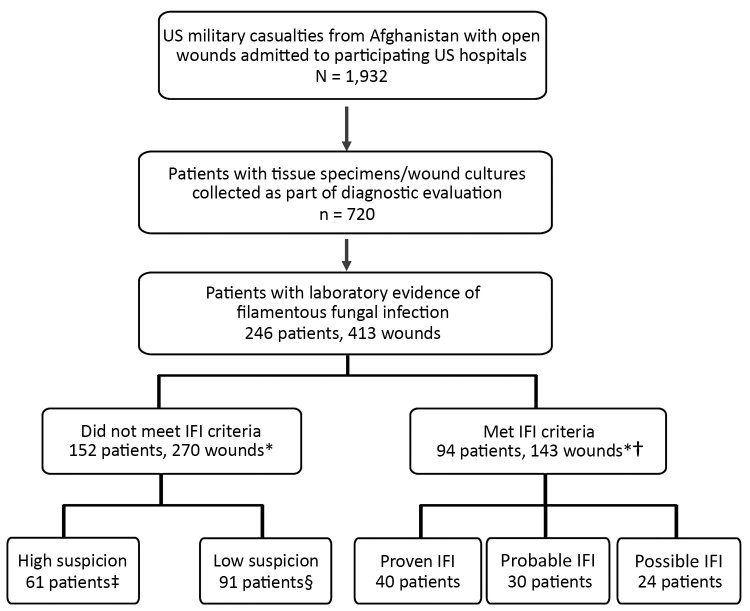
Combat casualties with laboratory evidence of fungal infection in study of US military patients who had laboratory evidence of fungal infection after battlefield trauma in Afghanistan, June 1, 2009–December 31, 2014. *Total of 143 IFI wounds, 120 high-suspicion wounds, and 150 low-suspicion wounds. For the person-level analysis, patients with multiple wounds were included in the IFI group even if 1 of their wounds met criteria other than for an IFI; similarly, patients with both low-suspicion and high-suspicion wounds were included in the high-suspicion group. †94 patients had 143 wounds that met criteria for an IFI; these same patients had 31 wounds that met criteria for high-suspicion wounds and 16 wounds that met criteria for low-suspicion wounds. ‡61 patients had 89 wounds that met criteria for high-suspicion wounds and 14 wounds that met criteria for low-suspicion wounds. §91 patients had 120 wounds classified as low-suspicion wounds. IFI, invasive fungal infection.

Of the 1,932 patients evaluated at the participating hospitals, 720 (37%) had penetrating wounds and operative cultures/histopathology findings submitted for evaluation. Of these, 246 (34%) had >1 wound with laboratory evidence of fungal infection (Figure 1). All patients were young men; median age at injury was 24 years (interquartile range [IQR] 21–27 years). Nearly all patients had been injured by a blast (98%) while on foot patrol (95%).

The 246 patients had 413 wounds with laboratory evidence of a filamentous fungus. Of these, 143 wounds (94 patients) met the criteria for an IFI (Figure 1). The remaining 270 wounds did not meet IFI criteria, either because they appeared no longer suspicious for infection (i.e., no ongoing necrosis after >2 debridements; 167 wounds) or because laboratory evidence of fungal infection was evident early (i.e., at or before the first or second debridement) but not in subsequent samples (103 wounds). Of the 270 non-IFI wounds, 194 met criteria for deep SSTI; 120 of those were treated with directed antifungal therapy and were classified as high-suspicion wounds (107 with >10 days of antifungal therapy and 13 with <10 days of antifungal therapy but the patient had undergone a proximal amputation or died). Of the 150 low-suspicion wounds, 76 did not meet criteria for deep SSTI and 74 met the criteria for deep SSTI but were treated with either directed antibiotic therapy (36 wounds) or antifungal therapy for <10 days (38 wounds). Most wounds classified as low suspicion had only positive culture results (125 [83.3%] wounds), and none showed angioinvasion histopathologically.

### Proven, Probable, and Possible IFI

Patients with proven, probable, and possible IFIs had been critically injured, and most had an ISS of >26: 98% in the proven group, 90% probable, and 88% possible (Table 2). SOFA scores at admission to US hospitals were lower among those in the possible IFI group (p = 0.007). Otherwise, no clinically relevant distinguishing differences were found among the IFI classification groups. Patients with IFI classified as proven or probable had received antifungal therapy longer than those with possible IFI (p<0.001; Table 2).

**Table 2:** Characteristics of US military patients with IFI after battlefield trauma in Afghanistan, June 1, 2009–December 31, 2014*

Characteristic	IFI			p value
	Proven, n = 40	Probable, n = 30	Possible, n = 24	
Blast injury	40 (100)	29 (96.7)	23 (95.8)	0.327
Injured while on foot patrol†	29 (100)	26 (96.3)	18 (85.7)	0.062
Injury severity score				
Median (IQR)	42 (33–57)	40 (33–50)	35 (30–44)	0.127
>26/critical	39 (97.5)	27 (90.0)	21 (87.5)	0.279
Blood units received 24 h after injury‡				
Median (IQR)	31 (23–43)	34 (23–47)	27 (17–37)	0.276
10–20 units	6 (15.0)	4 (13.3)	8 (34.8)	0.121
>20 units	33 (82.5)	24 (80.0)	14 (60.9)	0.074
Traumatic amputation§	30 (75.0)	20 (66.7)	14 (58.3)	0.376
SOFA score, median (IQR)				
Germany	11 (8–15)	10.5 (7–12)	11 (5–12)	0.413
US hospital	9 (5–13)	7.5 (1–11)	4.5 (1–7.5)	0.007
Duration of antifungal use, median (IQR)	36 (23–49)	24 (18–36)	16 (0–24)	<0.001
Outcome				
Surgical amputations¶	27 (67.5)	13 (43.3)	10 (41.7)	0.057
Death	7 (17.5)	1 (3.3)	0	0.030

*Values are no. (%) except as indicated. IFI, invasive fungal wound infections; IQR, interquartile range; SOFA, sequential organ failure assessment. †Status of whether patient was on foot patrol or in a vehicle is missing for 17 IFI patients (11 Proven, 3 Probable, and 3 Possible). Percentages and p-values based on total minus missing. ‡Blood information is missing for 1 patient with a possible IFI. Percentages and p-values based on total minus missing. §Includes amputations that occurred before admission to a US hospital. ¶Defined as amputations that occurred after admission to a US hospital.

### IFI, High-Suspicion, and Low-Suspicion Wounds

Patient demographics and mechanisms of injury were similar among those with IFI, high-suspicion, or low-suspicion wounds. Injury severity was high overall (median ISS 34, IQR 30–45). Thus, a large proportion of patients with wounds classified in all 3 groups had undergone amputations (68% for IFI, 79% for high suspicion, 80% for low suspicion; Table 3).

**Table 3:** Characteristics of US military patients with laboratory evidence of invasive fungal infection of wound sustained on battlefield, Afghanistan, June 1, 2009–December 31, 2014*

Characteristic	IFI, n = 94	High suspicion, n = 61	p value†	Low suspicion, n = 91	p value‡
Blast injury	92 (97.9)	61 (100)	0.520	89 (97.8)	1.000
Injured while on foot patrol§	73 (94.8)	51 (94.4)	1.000	80 (95.2)	1.000
Injury severity score					
Median (IQR)	40 (33–50)	38 (30–45)	0.262	33 (27–42)	<0.001
≥26/critical	87 (92.6)	52 (85.3)	0.144	75 (82.4)	0.037
Blood units received 24 h after injury, median (IQR)¶	31 (21–43)	21 (15–32)	0.003	17 (12–24)	<0.001
10–20	18 (19.4)	25 (41.0)	0.003	42 (48.3)	<0.001
>20	71 (76.3)	31 (50.8)	0.002	30 (34.5)	<0.001
Traumatic amputation#	64 (68.1)	48 (78.7)	0.150	73 (80.2)	0.060
SOFA score, median (IQR)					
Germany	11 (7–13)	8 (4–13)	0.028	6 (2–9)	<0.001
US hospital	7 (2–11)	4 (1–8)	0.022	1 (0–6)	<0.001
Duration of antifungal use, median (IQR)	24 (14–43)	21 (14–27)	0.006	0	NA
Outcome					
Surgical amputation**	50 (53.2)	26 (42.6)	0.199	24 (26.4)	<0.001
Death	8 (8.5)	1 (1.6)	0.090	0	0.007

*Values are no. (%) except as indicated. Patients with >1 wound with differing classifications are classified at the highest level. One patient with a wound classified as high suspicion died within 24 h of collection of sample providing laboratory evidence of fungal infection, precluding classification as having an IFI. IFI, invasive fungal wound infection; IQR, interquartile range; SOFA, sequential organ failure assessment. †Compares characteristics between those having an IFI and those having a high-suspicion wound. ‡Compares characteristics between those having an IFI and those having a low-suspicion wound. §Information about whether patient was on foot patrol or in a vehicle is missing for 17 IFI patients, 7 patients with high-suspicion wounds, and 7 patients with low-suspicion wounds. Percentages and p-values based on total minus missing. ¶Information missing for 1 patient with an IFI and 4 patients with low-suspicion wounds. Percentages and p-values based on total minus missing. #Includes amputations that occurred before admission to a US hospital. **Defined as amputation that occurred after admission to a US hospital.

### IFI and High-Suspicion Wounds

For patients with IFI and high-suspicion wounds, ISSs were similar (median 40 vs. 38; p = 0.262; Table 3). Compared with patients with high-suspicion wounds, IFI patients had higher SOFA scores at admission to LRMC (median 11 vs. 8; p = 0.028) and US hospitals (median 7 vs. 4; p = 0.022) and received more blood transfusions within 24 hours of injury (median 31 vs. 21; p = 0.003). Patients with wounds classified as IFI also received antifungal therapy longer than patients with high-suspicion wounds (median 25 vs. 21; p = 0.006). Although blood urea nitrogen levels differed significantly between the groups, the levels were not clinically meaningful (data not shown). When analysis was restricted to the 56 patients with wounds that only met IFI criteria only or the 50 patients whose wounds only met high-suspicion criteria, IFI patients were more likely to have received >20 units of blood within 24 hours (70% vs. 58%; p = 0.016).

### IFI and Low-Suspicion Wounds 

The median ISS was higher among patients with IFI wounds than among those with low- suspicion wounds (40 vs. 33; p<0.001; Table 3). Compared with patients with low-suspicion wounds, patients with IFI wounds had higher SOFA scores at admission to LRMC (11 vs. 6; p<0.001) and to US hospitals (7 vs. 1; p<0.001) and received more blood transfusions within 24 hours of injury (median 31 vs. 17; p<0.001). Blood urea nitrogen levels, liver function test results, and leukocyte counts were also higher among those in the IFI group (data not shown). Patients classified as having IFI also received antifungal therapy longer than those with low-suspicion wounds (median 25 vs. 0; p = 0.006). When analysis was restricted to patients whose wounds only met IFI criteria (56) or patients whose wounds only low-suspicion criteria (91), statistical differences were similar to those of the full population.

### Wound Microbiology

**Figure 2:**
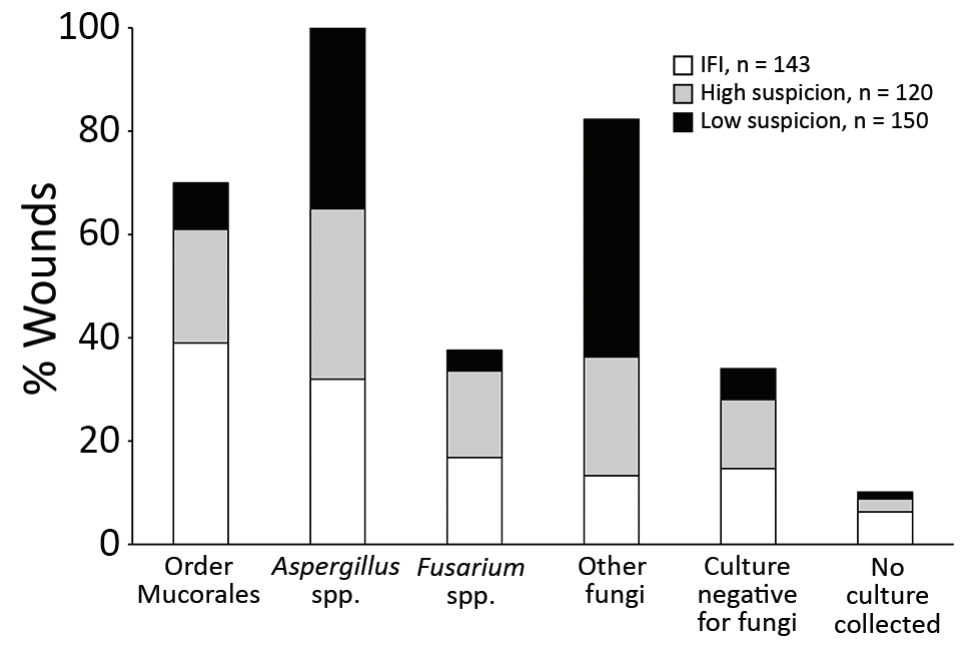
Wound culture mycology distribution, by wound classification, in study of US military patients who had laboratory evidence of fungal infection after battlefield trauma in Afghanistan, June 1, 2009–December 31, 2014. Because wound infections were polymicrobial, organisms are not mutually exclusive for a classification type. IFI, invasive fungal infection; other fungi, filamentous fungi other than order *Mucorales, Aspergillus* spp., and *Fusarium* spp.

Among 413 wounds with documented laboratory evidence of fungal infection, fungal cultures had been submitted for 97% and culture results were negative for 11% (Figure 2; Table 4). Fungi of the order Mucorales were more likely to be isolated from IFI wounds (39%) than from high-suspicion (22%) and low-suspicion (9%) wounds (p<0.05; Table 4). Fungi of the order Mucorales were isolated from over half (52.6%) of IFI wounds with documented angioinvasion (proven IFI), 31.3% of probable IFI wounds, and 26.3% of possible IFI wounds (p = 0.016). In contrast, a higher proportion of low-suspicion (46%) and high-suspicion (23%) than IFI (13%) wounds grew other fungi (p<0.05). Fungi belonging to the genus *Fusarium* were more commonly isolated from IFI wounds than from low-suspicion wounds (17% vs. 4%; p<0.001). Between high-suspicion and low-suspicion wounds, the proportions of growth of fungi of the order Mucorales (p = 0.003), *Fusarium* spp. (p = 0.001), and other fungi (p<0.001) differed significantly.

**Table 4:** Microbiological findings for US military patients who had battlefield trauma wounds with invasive fungal infections and laboratory evidence of fungal infection, June 1, 2009–December 31, 2014*

Culture findings	IFI wounds, n = 143	High-suspicion wound, n = 120	p value†	Low-suspicion wound, n = 150	p value‡
Fungal cultures not sent	9 (6.3)	3 (2.5)	0.235	2 (1.3)	0.032
Fungal growth§					
None	21 (14.7)	16 (13.5)	0.774	9 (6.0)	0.014
1 fungus	55 (38.5)	50 (41.7)	0.597	91 (60.7)	<0.001
>1 fungi	58 (40.6)	51 (42.5)	0.751	48 (32.0)	0.128
>1 fungi plus bacteria*¶*	82 (57.3)	80 (66.7)	0.121	83 (55.3)	0.729
Order Mucorales	55 (38.5)	26 (21.7)	0.003	13 (8.7)	<0.001
*Aspergillus* spp.	45 (31.5)	39 (32.5)	0.858	55 (36.7)	0.348
*Fusarium* spp.	24 (16.8)	20 (16.7)	0.980	6 (4.0)	<0.001
Other filamentous fungi#	19 (13.3)	27 (22.7)	0.046	69 (45.7)	<0.001
Bacterial growth§					
None	3 (2.1)	1 (0.8)	0.628	3 (2.0)	≈1.00
Staphylococcus aureus**	0	0	NA	2 (1.3)	0.499
*Enterococcus* spp.	53 (37.1)	51 (42.5)	0.369	42 (28.0)	0.098
* E. faecalis*	5 (3.5)	5 (4.2)	0.777	8 (5.3)	0.445
* E. faecium*	41 (28.7)	42 (35.0)	0.271	31 (20.7)	0.111
* Escherichia coli*	22 (15.4)	20 (16.7)	0.777	23 (15.3)	0.990
*Pseudomonas* spp.	21 (14.7)	23 (19.2)	0.332	16 (10.7)	0.301
* P. aeruginosa*	16 (11.2)	14 (11.7)	0.903	11 (7.3)	0.254
* Acinetobacter baumannii*	29 (20.3)	11 (9.2)	0.012	6 (4.0)	<0.001
Other gram-negative bacilli	30 (21.0)	29 (24.2)	0.537	21 (14.0)	0.115
ESKAPE pathogen††	49 (34.3)	50 (41.7)	0.217	44 (29.3)	0.365
Multidrug resistant‡‡	53 (37.1)	34 (28.3)	0.134	26 (17.3)	<0.001

*Values are no. (%) except as indicated. IFI, invasive fungal infection; NA, not applicable. †Compares characteristics between IFI and high-suspicion wounds. ‡Compares characteristics between IFI and low-suspicion wounds.  §Because of polymicrobial wounds, organisms are not mutually exclusive and will add to more than the total. Bacterial cultures were restricted to those collected within 14 d of injury. ¶Category of >1 fungi plus bacteria is not mutually exclusive from fungal cultures with 1 fungus or >1 fungi. #Includes *Acrophialophora* spp., *Alternaria* spp., *Bipolaris* spp., *Scedosporium* spp., and *Trichoderma*. **Includes methicillin-resistant and methicillin-susceptible *S. aureus.* ††ESKAPE pathogens are *Enterococcus faecium*, *Staphylococcus aureus*, *Klebsiella pneumoniae*, *Acinetobacter baumannii*, *Pseudomonas aeruginosa*, and *Enterobacter* spp. ‡‡Multidrug resistant is defined as resistance to ≥3 of 4 antibiotic classes or producion of extended-spectrum β-lactamase or carbapenemases.

Bacterial cultures collected within 14 days of injury were assessed for 378 (92%) of wounds; only 1% were negative for bacteria (Table 4). Most frequently isolated were *Enterococcus* spp. (38%) and *Escherichia coli* (17%); not commonly isolated was *Staphylococcus aureus* (0.5%). The bacteria isolated differed among the 3 groups of wounds. *Acinetobacter baumannii* was more frequently isolated from patients in the IFI group (22%) than from patients in the other 2 groups (9% with high-suspicion wounds [p = 0.006] and 5% with low-suspicion wounds [p<0.001]). In addition, the proportion of multidrug-resistant organisms isolated was higher among patients with IFI (37%) than among those with low-suspicion (17%) wounds (p<0.001).

### Outcomes

The proportion of deaths or surgical amputations did not differ significantly between patients in the IFI and high-suspicion groups. A higher proportion of patients in the IFI group required a surgical amputation (53% vs. 26%; p<0.001) or died (9% vs. 0; p = 0.007) than did patients in the low-suspicion group (Table 3). The number of debridements in the first 4 weeks after injury was similar for patients with IFI wounds (median 10, IQR 7–11) and high-suspicion wounds (median 9, IQR 7–11; p = 0.034); however, patients with low-suspicion wounds underwent fewer debridements (median 7; IQR 5–9; p<0.001) than patients with IFI wounds. In addition, 70 (49%) of 143 IFI wounds required that the patient undergo surgical amputations compared with 48 (40%) of 120 high-suspicion wounds (p = 0.146) and 45 (30%) of 150 low-suspicion wounds (p = 0.001).

## Discussion

As part of our comprehensive evaluation of IFIs among US military personnel wounded in Afghanistan, we propose definitions for the risk stratification of wounds with laboratory evidence of fungal infection (i.e., positive culture results, histopathologic results, or both). Using our definitions, wounds can be grouped into 3 relatively homogeneous groups with different probabilities of IFI: wounds with IFI, those at high suspicion for IFI, and those at low suspicion for IFI. The categorization of wounds into risk groups is designed to provide a framework to help with clinical decision making and reduce practice variations and to provide definitions that could be used to group wounds for clinical and epidemiologic research.

We had previously proposed a modification of the EORTC/MSG criteria (*18*) to provide a better disease definition and classification for trauma-related IFI. Using this definition, wounds with necrosis present after >2 debridements and laboratory evidence of filamentous fungi at any time (either early or late) were classified as IFI (*1*,*3*). We had previously considered this classification sufficient for IFI wounds in the military setting and for clinical care (*1*,*3*). However, a comprehensive review of all cases suggested that the previously proposed criteria failed to sufficiently account for temporal aspects relevant to fungal contamination of wounds and necrosis associated with trauma. 

In this iteration, to account for the temporality of events, we propose a definition that requires ongoing necrosis and persistence of laboratory evidence of fungus after >2 debridements to define IFI. Furthermore, we categorize wounds into 3 categories of varying risk, whereas the previous definition defined only IFIs. The prior definition was based on review of 37 initial cases, whereas this definition is comprehensive and includes data from 246 patients. Were we to apply the current criteria to 77 previously identified cases (*3*), one third of them would no longer be classified as IFI (25% would be reclassified as high suspicion and 10% as low suspicion; data not shown).

According to our schema, IFI case-patients accounted for ≈5% (range 2.7%–6.6% annually) of all admissions for battlefield trauma during 2009–2014; however, it should be noted that specimens were not consistently collected for histopathologic examination until late 2010. Of 94 IFI patients, 8 (9%) died and about half underwent surgical amputations. Two thirds of wounds with laboratory evidence of fungal infection did not meet our definition of IFI. Specifically, one third of wounds were classified as low suspicion, and patients with these wounds generally had very severe injuries; however, by the time of admission to a US hospital, they were not critically ill, as evidenced by a median SOFA score of 1, and they were less likely to undergo subsequent surgical amputation. Laboratory evidence for these patients was often based on isolation of fungi (83%) with negative histopathologic findings. These patients were also less likely to receive antifungal medications (only 15%) and to receive them for a shorter duration (median 6.5 days for patients with low-suspicion wounds who received antifungal medications). Among this group, approximately half of the wounds had no evidence of deep SSTI, which confirms that isolation of fungus from a wound, even in critically injured patients with blast injuries sustained on foot patrol, is not enough evidence to suggest an IFI (*13*).

Given the substantial morbidity associated with IFI, in February 2011, a hospital-based clinical practice guideline was implemented at LRMC to enable earlier IFI diagnosis and initiation of antifungal therapy with the goal of improving clinical outcomes. Per the guideline, based on previously identified independent risk factors, specimens for histopathologic examination and fungal/bacterial cultures were systematically collected from at-risk patients after the first wound debridement (*11*). Although this risk-based sampling strategy successfully resulted in earlier IFI diagnosis (average 4 vs. 9 days before implementation of the guideline), it also resulted in practice variation and use of antifungal medications for patients with low risk for wound progression to IFI (*11*). Our study suggests that approximately one third of patients for whom tissue was submitted had laboratory evidence of a fungus. Therefore, it became essential to objectively discriminate between wounds that require intensified surgical management and initiation of antifungal medications and wounds that can be closely followed up without substantial interventions (*13*). Because this patient group was composed primarily of men critically injured in a blast while on foot patrol and who received massive blood transfusions, all risk factors for IFI (demographic characteristics and injury patterns), although useful for identifying those at risk for an IFI (*5*), do not discriminate among those who need intensified surgical management and antifungal medications and those for whom antifungal medications can be withheld. Similarly, laboratory parameters differed significantly but are not clinically meaningful. Thus, we examined and used wound characteristics (i.e., persistence of necrosis, local signs and symptoms of a deep SSTI) in our classification. Empiric use of antifungal medications was common in this population (received by 63%); similarly, isolation of bacteria was very common (98% of cultures). Hence, to try and delineate between bacterial and fungal wound infections, we incorporated the prolonged use of antifungal medications (>10 days) in our classification schema. Although this measure is based on the provider’s judgment, we believe that the focus on local signs and symptoms of a wound, along with wound mycology, can be used for clinical decision making; however, our classification needs to be validated prospectively in other civilian and military trauma settings, outside of the Afghanistan theater, and ideally prospectively. 

On the basis of IFI risk factors, a tool to support clinical treatment decisions near the point of injury and after admission to military hospitals has been developed (*20*). Data from our analysis may be used to further refine that clinical tool. The Joint Trauma System provides evidence-based recommendations for trauma care for the military. The Joint Trauma System has developed a Clinical Practice Guideline for management of IFI in wounded persons (*21*), and data from this analysis have been briefed to the Joint Trauma System leadership for potential refining of the IFI guidelines to enable wider dissemination throughout the military care community.

Clinical mycology, although not used in our classification schema, is another feature for distinguishing wounds. Fungi of the order Mucorales (39%) predominated in IFI wounds, and other fungi were more frequent in low-suspicion wounds (46%). The negative effect that fungi associated with IFIs have on wound healing has been previously demonstrated; fungi from the order Mucorales are associated with a statistically significant longer time to wound closure (*12*). Thus, when ongoing necrosis, persistence of laboratory evidence of fungus, and objective evidence of deep SSTI are lacking, antifungal medications can be withheld if the patient is closely followed. In particular, antifungal medications may be withheld when high-risk features such as growth of order Mucorales fungi or angioinvasion are lacking.

In conclusion, we found that blast-associated injuries were common in this population of US service members and resulted in multiple heterogeneous wounds with evidence of fungal infection. Focusing on the wound characteristics (e.g., absence of ongoing necrosis and persistence of fungi), especially in the absence of objective signs of deep SSTIs, identifies wounds at low risk for IFI. When close clinical follow-up can be ensured, these wounds can be monitored without the immediate use of antifungal therapy. The characteristics of the fungi isolated also seem to stratify wound risk; isolation of fungi of the order Mucorales is associated with wounds with IFI or highly suspicious of IFI. Our proposed definitions help divide wounds into 3 groups based on the certainty of diagnosis, providing a framework to support clinical decision making, both initial empiric and subsequent targeted antifungal therapy, and reductions in practice variations.
